# Healthcare professionals’ discussion of loss and grief with parents of children with life-limiting severe neurological impairment: Findings from a scoping review

**DOI:** 10.1017/S1478951524001743

**Published:** 2025-02-21

**Authors:** Elaine Brennan, Marian (Mya) Clarke, Suzanne Guerin

**Affiliations:** UCD School of Psychology, University College Dublin, Dublin, Ireland

**Keywords:** Loss and grief, parents, child, severe neurological impairment, healthcare professionals

## Abstract

**Objectives:**

Parents’ experiences of loss and grief in the context of caring for a child with life-limiting severe neurological conditions are complex. Supportive interventions delivered by multidisciplinary teams have the potential to mitigate illness-related and anticipatory grief before and after bereavement. To date, the literature on professionals’ discussion of loss and grief with parents has not been synthesized. This systematic review aims to synthesize the evidence to establish what is known about professionals’ experience of these discussions with this population, with particular emphasis on timing, frequency, and the setting in which discussions occur.

**Methods:**

A scoping review was developed, informed by the Preferred Reporting Items for Systematic Reviews and Meta analyses – Scoping Extension guidelines and the PCC (Population, Concept, Context) framework. Three electronic databases (PsycINFO, CINAHL, and PubMED) were searched using medical subject heading (MeSH) terms and keywords search strings in January 2023. The search was not limited to year of publication. Overall, 35 articles were analyzed using a combination of descriptive analysis and thematic synthesis.

**Results:**

Two overarching themes were identified, “loss and grief are part of this context” and “lack of recognition of loss and grief,” illustrating that despite the lack of evidence of explicit discussion of these issues, some aspects of loss and grief appeared to guide or implicitly influence healthcare professionals’ practice. Failure to acknowledge loss and grief was associated with an increase in parental distress and had implications for future care planning.

**Significance of results:**

Healthcare professionals are well placed to discuss loss and grief with parents of children with life-limiting severe neurological conditions. However, these discussions are only implicitly reported in the literature. Findings suggest that some professionals avoided discussing loss and grief. Bereavement outcomes are not typically considered in findings of the papers reviewed. Based on these findings, future research should focus on what this means for understanding professionals’ capacity to engage with loss and grief.

## Introduction

Globally, estimates indicate that 8 million children per year live with some form of life-limiting condition for which there is no hope of cure and from which a child or young person will die (Connor et al. 2017; Together for Short Lives 2017). Of these, children with severe neurological impairment (SNI) form a distinct and diverse population (Allen et al. 2020). Common features of associated conditions include severe motor and cognitive impairments with co-occurring medical complexity (Cohen et al. 2011). Due to medical and technological advances, growing numbers of affected children now survive with lengthier trajectories (Koch and Jones 2018). These children require intensive caregiving and assistance with activities of daily living (Clarke and Quin 2007). The illness course typically follows a downward trajectory, with periods of relative stability interspersed with episodes of acute ill-health as the condition progresses (Steele 2000). Increasingly, it is recognized that good pediatric palliative practice involves caring for parents and assessment of the family situation (Koch and Jones 2018). Recent literature reveals the complex pervasive grief experienced relating to the loss of previously sustaining world assumptions, the wished for child, and inevitable, their child’s death. This type of loss is characteristically disenfranchised (Doka 2002), falling outside normative language and customs of death-related grief. Indeed, parents may struggle to express emotions or fully comprehend their loss experience (Neimeyer and Krawchuk 2020).

However, current theoretical models of grief provide a strong empirical basis on which to approach the care of bereaved individuals (Bonanno et al. 2002; Coleman and Neimeyer 2010; Klass et al. 2014; Stroebe and Schut 2010). Notably, newer developments in our understanding of grief incorporate the experience of significant non-death losses and emphasize the significance of meaning reconstruction in the grieving process (Braun and Berg 1994) and have moved away from stage or task model approaches (Parkes 1988; Worden 2009). In bereavement, significant losses are shaped by the social environment where interpersonal interactions influence how we grieve. Concepts such as meaning-making, resilience, and identity change have significance for how we relearn the world after loss (Bonanno 2009; Lichtenthal et al. 2010; Neimeyer et al. 2014). Studies show that finding meaning in loss helps individuals move forward with life, create new meaning, and build capacity for the future (Lichtenthal et al. 2010). In palliative care, the absence of meaning-making care has been shown to predict difficulties with anticipatory grief, and complicated grief symptoms after the death occurs (Burke et al. 2019). In this regard, parents who experience distress associated with rumination about events during the pregnancy or birth may find telling their story to a family member or professional helpful for reaching some consoling explanation for their child’s condition. Over time, communication about important areas of palliative practice, such as goals of care, decision-making, and advance care planning requires professionals to feel comfortable with loss and grief. Studies of pediatric advance care planning have found that avoidance results in later conversations not being beneficial for parents and professionals (Carr et al. 2021).

Living with the prospect of losing a child is regarded as one of the most stressful and traumatic experiences for parents (Janoff-Bulman 2010; Rando 1986). Moreover parents of children with SNI live in a state of constant vigilance for signs of the next crisis, with the care journey described as an emotional rollercoaster (Rallison and Raffin-Bouchal 2013). In bereavement literature, intensive caregiving has been found to place parents at greater risk of morbidity, mortality, and prolonged grief disorder after the death occurs (Li 2003). Although studies have found that outcomes are influenced by experiences during the illness, the end-of-life phase, and the circumstances of the death (Keesee et al. 2008; Kriecberg et al 2007; Wijngaards-de Meij et al. 2008a), gaps remain in our understanding of anticipatory grief. At the same time, a hopeful outlook may present a challenge to professionals seeking to prepare parents for the inevitability of death. To date, interventions developed by healthcare professionals to support parents to deal with loss have tended to focus on bereavement care after the death while pediatric palliative care research has principally focused on alleviating concerns about quality of life during the end-of-life phase (Mack and Wolfe 2006; Ribbers et al. 2020).

This scoping review aims to synthesize what is known about healthcare professionals’ discussions of loss and grief with parents of children with life-limiting severe neurological impairment (LLSNI) to establish how these issues are considered, conceptualized, and understood within current literature. Identifying the approach, timing, frequency, and setting in which these discussions occur has implications for practitioners.

## Study design

Scoping reviews are useful within health and social sciences disciplines when the aim is to seek conceptual clarification by mapping existing research prior to embarking on further research (Khalil et al. 2016). The methods employed in this review align with methodological developments since the framework was originally established by Arksey and O’Malley (2005) and included an *a priori* protocol (Khalil et al. 2016) which was registered on the Open Science Framework on 8 March 2023 (10.17605/OSF.IO/ZEF5W) and can be accessed at https-//archive.org/details/osf-registrations-zef5w-v1. The review was developed and conducted in accordance with the Preferred Reporting Items for Systematic Reviews and Meta analyses – Scoping Extension (PRISMA-ScR) checklist (Tricco et al. 2018).

The PCC mnemonic (population, concept, fcontext) guided the construction of the research question, objectives, and eligibility criteria, and informed the search strategy (Khalil et al. 2016). During the early stages of the review, the definition of medical conditions to be included was reviewed and refined. Ultimately, we chose to use the term children with SNI, as this best describes the population of interest, using Allen et al.’s definition as follows; “Severe neurological impairment describes a group of disorders of the central nervous system which arise in childhood, resulting in motor impairment, cognitive impairment and medical complexity, where much assistance is required with activities of daily living. The impairment is permanent but can be progressive or static” (Allen et al. 2020, p. 85). The population was defined as parents of children with a life-limiting SNI, the concept as professionals’ discussion of loss and grief, while the context refers to the timing, frequency, and settings in which discussions occurred.

## Eligibility criteria

The search was exhaustive, using all years of records and countries of publication. Due to resource limitations, the search was confined to articles published in the English language. Children were defined up to the age of 18 in accordance with the UN Convention on the Rights of the Child (UN General Assembly 1989). Table 1 outlines the inclusion and exclusion criteria.

**Table 1.** Inclusion and exclusion criteria informing the search

Inclusion criteria	Exclusion criteria
1. Articles reporting studies relating to children with life-limiting severe neurological impairment (LLSNI) in line with the definition proposed by Allen et al. 2020. 2. Articles reporting discussions of loss and grief by health care professionals with parents of children with LLSNI at the time of diagnosis and throughout the child’s lifespan. 3. Articles reporting on discussions about advance care planning or end-of-life planning where there is an implicit awareness of aspects of loss and grief. 4. The study reports on the views of professional carers or the views of parents reporting their engagement with health care professionals on this issue. 5. Studies that include parents of children with other life-limiting conditions if the findings relating specifically to children with LLSNI can be extracted and synthesized separately or if the majority of the sample meet the first criterion. 6. Articles reporting primary, empirical research. 7. Research articles published in peer-reviewed journals in the English language. 8. Children are defined as persons from birth to 18 years.	1. Articles reporting secondary research (e.g., systematic reviews, meta-analyses), conference abstracts, theoretical/conceptual papers, book chapters (unless peer-reviewed and presenting primary empirical research), opinion pieces, protocols. 2. Articles reporting discussions by healthcare professional with parents of children with life-limiting diagnoses that do not explicitly report on discussions with parents of children with LLSNI. 3. Studies reporting on health care professional discussions with parents of children over the age of 18 years.

## Search strategy

Keywords and index terms were initially identified through preliminary searching in the title and abstract of existing reviews that were relevant to the current review across 2 databases (Cooper et al. 2018). Searching these reference lists identified key articles that were then used to gather synonyms and terminology for the final search string. Boolean operators and truncation were used in each database to efficiently identify all relevant findings. Trial searches were conducted repeatedly, and adjustments made during the months of November and December 2022 to validate the search string by identifying these same key articles in the results of the search. An academic librarian was consulted throughout this process. The scope of the search extended beyond articles specifically focused on professional discussion of loss and grief to include papers that referenced this as part of a wider discussion. For this reason, the term “professional discussion” was not used as a search term and was incorporated in the inclusion criteria instead. The final keywords used included variants across the 4 terms child, parent, life-limiting neurodevelopmental disability (previous descriptor for SNI) and grief, with a sample search string developed for use in PsycINFO provided in supplementary material.

## Sources of evidence

The electronic databases PsycINFO, CINAHL, and PubMed were chosen for their relevance to psychology, social sciences, nursing, and medical research. All final searches were run in January 2023 and imported to Endnote, with duplicates removed and final records uploaded to Covidence for screening. A pilot title and abstract screening of the first 30 records generated by the PsycINFO database was undertaken by 2 reviewers (EB & SG) to test and refine the eligibility criteria. As a notable number of studies reported on findings from mixed populations, the inclusion criteria were broadened to include articles where findings could be separately extracted. The remaining records were independently screened by title and abstract by the first 2 authors (EB & MC) and disagreements resolved by the third author (SG). Review and selection of full text studies that met the inclusion criteria was undertaken independently by the first author and a researcher (EB & PS), and any disagreements were resolved as above. At this stage, the reason for exclusion was recorded. Finally, reference lists of included full text studies were hand-searched for relevant articles and any records identified subjected to the same screening process.

## Data extraction and synthesis

Relevant data from each of the included articles were extracted into a template by the first author (EB), including information on author, year of publication, study aims, methodology, population, key findings, and study limitations. At the same time, quality appraisal of the articles was conducted using the Mixed Methods Appraisal Tool (Hong et al. 2018). The extraction was piloted on 5 studies to ensure the process was effective and all relevant information extracted. Following a review of this sample, data extraction was completed by the first author.

All quantitative data were converted to qualitative data prior to analysis. Qualitative thematic synthesis (Thomas and Harden 2008) was used to generate descriptive and interpretative themes. Following data familiarization, 2 reviewers (EB & SG) independently assigned open codes to a sample of 5 articles. The reviewers then compared labels assigned to agree a set of codes going forward. All articles were coded in this way to develop a coding framework. Similar codes were then grouped into categories to produce a descriptive analysis of relevant data related to professionals’ discussion of loss and grief. It was noted that none of the papers identified contained explicit discussions of loss and grief between parents and professionals. However, there was clear discussion of aspects of death and dying, and 2 broad themes were developed from the analysis of these discussions: “Loss and grief is part of this context” and “Lack of recognition of loss and grief.”

## Results

**Figure 1.**
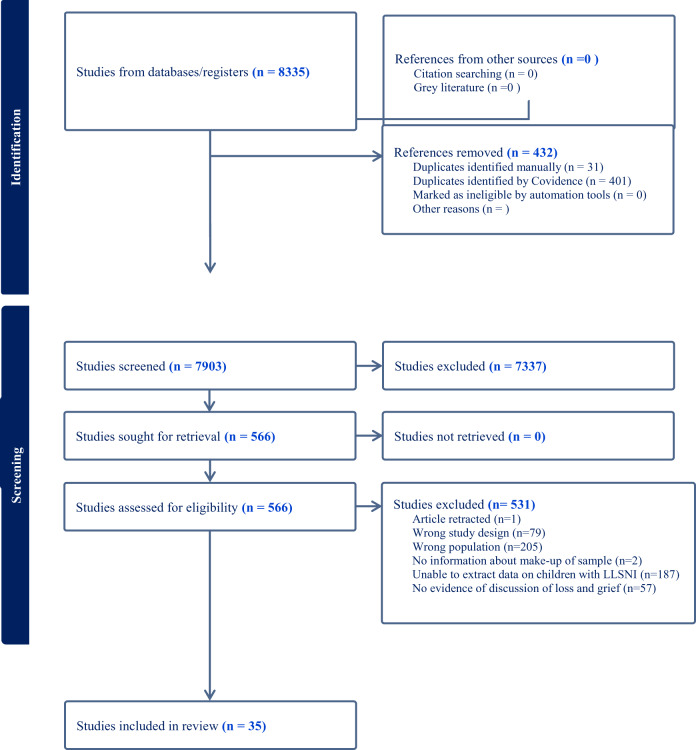
PRISMA flowchart documenting the search process and outcomes.

In total, 35 papers were identified that met the inclusion criteria, with 34 papers included in the final analysis. The search process is represented in Figure 1, while the key details from the 35 papers are presented in Table 2. One study produced 2 papers (Janvier 2016; Janvier et al. 2020), 1 produced 3 papers (Bogetz et al. 2022; Bogetz et al. 2021; Bogetz et al. 2020) and a third (Sullivan et al. 2015, 2019, 2020, 2014) 4 papers. In 25 papers, the participants were parents (See Table 2), in 7 they were professionals, and the remaining 3 combined both parents’ and professionals’ perspectives. In terms of the assessment of quality, the MMAT was applied to all papers. One paper failed to meet the minimum criteria (based on the screening questions) and was excluded. For the remaining papers, there were no methodological areas of concern in the body of research.

**Table 2.** Summary of studies included themes and illustrative findings

Study ID	Authors Country	Study aim	Research design	Analysis	Key themes and illustrative findings	MMAT criteria applied
1	Bao et al. (2021) Canada and U.S.	To describe patient characteristics, symptoms, use of medications, discussion of resuscitation orders, and care provided preceding and during the end of life.	Mixed methods	Descriptive analysis	**Theme 1:** Findings suggested that the care provided for most children at the time of death was supportive and involved palliative care. **Theme 2:** A proportion of children may have received invasive treatments at the time of death.	Quantitative as findings focused on quantitative results.
2	Beecham et al. (2017) U.K. and USA	To explore parents’ experiences in the U.K. of ACP discussions for children for whom cure is not likely.	Qualitative	Grounded theory	**Theme 1:** Parents were involved in discussions and decision-making about place of care, place of death and the limitation of treatment. **Theme 2:** Some parents felt that professionals were overly focused on symptom management rather than understanding the needs of the whole family.	Qualitative
3	Beernaert et al. (2019) Denmark	To assess the experiences and wishes of parents of children with severe spinal muscular atrophy (SMA) regarding information and decision-making throughout the course of the illness.	Quantitative	Descriptive statistics	**Theme 1**: Most bereaved parents had their wishes honored about the location of their child’s death. **Theme 2:** Some parents reported they were not given all the prognostic information and treatment options.	Quantitative
4	Bogetz et al. (2020) U.S.	To explore aspects important to preparedness at EoL among bereaved parents of children with complex chronic conditions.	Quantitative	Thematic analysis	**Theme 1:** Findings suggested that parents coped with the deterioration in their child’s condition by normalizing their situation. **Theme 2:** Parents reported they felt responsibility for their child’s survival when end-of-life decisions were imposed on them.	Qualitative as findings focused on open-response survey items
5	Bogetz et al. (2021) U.S.	To identify supportive clinical care strategies that could be used to improve the care provided to children with complex chronic conditionss and their families.	Quantitative Open-response items from cross-sectional survey	Qualitative analysis	**Theme 1:** Parents identified caring professionals as those who recognized their expertise and experience as parents and remained involved into bereavement. **Theme 2:** Parents reported that doctors gave “false hope” by recommending multiple surgeries to prolong life but did not offer cure or improved quality of life.	Quantitative and Qualitative as statistical and thematic analysis used in results
6	Bogetz et al. (2022) U.S.	To retrospectively explore parents’ perspectives of children with SNI, on clinician counselling regarding home mechanical ventilation.	Qualitative	Inductive thematic analysis	**Theme 1:** Parents reported being upset when information about risk of death was shared insensitively. **Theme 2:** The use of intimidating examples as a communication technique.	Qualitative
7	Brecht & Wilkinson (2015) Australia	To describe the prevalence, nature and outcome of treatment limitation discussions (TLDs) in critically ill new-borns with severe brain injury.	Chart review	Descriptive statistics	**Theme 1:** Findings suggest that the majority of parents engaged in treatment limitation discussions with doctors. **Theme 2:** Some children whose parents had TLD’s survived, with no impairment, however this outcome was not included as part of these discussions.	Quantitative
8	Carr et al. (2022a) Ireland	To explore parents’ experience of the initiation of their child’s ACP discussions.	Qualitative	Reflexive thematic analysis	**Theme 1:** Parents reported they required care and support and for professionals to be aware of the individual family circumstances before engaging in ACP’s discussions. **Theme 2:** Parents reported that some professionals avoided discussions about the child’s EoL.	Qualitative
9	Carr et al. (2022b) Ireland	To identify types of professional behaviors associated with initiating advance care planning (ACP) discussions.	Quantitative cross sectional online survey	Descriptive and inferential statistics	**Theme 1:** Responding to parent cues was an important factor for initiating ACP discussions. **Theme 2:** Findings suggested that some professionals working in palliative care were uncomfortable discussing death.	Quantitative
10	Courtney et al. (2018) Ireland	The present study interviews mothers, exploring their experiences and the impact of caring onthemselves and their family.	Qualitative	Thematic analysis	**Theme 1:** Parents reported multiple losses and coped by normalizing their situation. **Theme 2:** Reports parents being given distressing information by doctors on their own. Parents described a pessimistic communication approach to diagnostic information sharing.	Qualitative
11	Currie et al. (2016) U.S.	How do bereaved parents describe their experiences related to their infant’s NICU hospitalization, end-of-life care, and palliative care consultation?	Qualitative	Qualitative content analysis	**Theme1**: Recognition of the importance of the parents’ role at end-of-life, e.g., allowing parent’s time to look after their baby after death. **Theme 2:** Parents reported that staffs were insensitive to their feelings of anticipatory grief when conflicting information was shared during the end-of-life stage.	Qualitative
12	Donovan et al. (2022) Australia	To ensure future service development and capacity building of health and social care professionals is informed by the lived experience of families whose child had received specialist PPC in Australia, supported through Quality of Care Collaborative Australia.	Qualitative	Inductive thematic analysis	**Theme 1:** PCC gave permission for “death talk” and encouraged parents to engage in memory making activities. **Theme 2:** Parents experienced further loss when staff who had been a witness to their loss and grief withdrew after the child’s death.	Qualitative
13	Edwards et al. (2012) U.S.	To investigate prior approaches to end-of-life planning for HMV patients with life limiting conditions, by reviewing the medical records of our deceased HMV patients and assessing the HMV program’s history of such preparation.	Quantitative chart review	Summary statistics based on predefined criteria	**Theme 1:** Findings suggest that professionals should **e**ncourage parents to contemplate goals when the child is stable as well as when the condition deteriorates. **Theme 2:** Findings suggest missed opportunities to engage in earlier EoL discussions.	Quantitative
14	Guerin et al. (2020) Ireland	To explore expert professionals’ opinions on service provision to children under 6 with a life limiting neurodevelopmental disability, including the goals of care and the integration and coordination of palliative care in general and specialist services.	Quantitative (Delphi method)	Ratings of agreement and consensus and nonparametric tests.	**Theme 1:** Professionals identified a number of changes which would improve coordination of services, including a single care plan and a keyworker. **Theme 2:** Parents were not viewed as experts in the child’s care, and professionals had poor knowledge about services available in the community. Palliative care was only considered late in the illness trajectory or during crisis management.	Quantitative
15	Guon et al. (2014) U.S.	The overall goal of this article is to gain a better understanding of parents who decided to continue their pregnancy after a PND of T13-18.	Mixed methods	Descriptive statistics and thematic analysis	**Theme 1:** There was evidence that professionals were sensitive to practice that could improve bereavement outcomes. **Theme 2:** The majority of parents considered some professionals did not value their child’s life and were focused on survival statistics. The majority of parents reported feeling under pressure to terminate their pregnancy.	Quantitative and qualitative
16*	Hammes et al. (2005) U.S.	Not stated by authors	Mixed methods	Chart review and content analysis	**Theme 1:** Parent reported advanced directive gave peace of mind. **Theme 2:** Focus on medical issues and no indication that psychosocial issues were being addressed.	Quantitative and qualitative as Mixed methods study
17	Heckford & Beringer (2014) U.K.	Our aim was to review advance care planning for children with life-threatening or life-limiting conditions (LTLLCs) in our local area	Quantitative	Case note review against 8 key goals advocated by ACT to promote optimal EoL planning.	**Theme 1:** Majority of children had written ACP. Most families were offered a choice about location of death. **Theme 2:** 25% of families had no documented advance care planning discussions. Where curative interventions were ongoing, professionals did not discuss the likelihood the child would die until child close to death.	Quantitative
18	Hurley et al. (2021) Ireland	To explore parental experiences surrounding the diagnosis of their child’s nonmalignant life-limiting condition.	Qualitative	Thematic analysis	**Theme 1:** Most parents recognized the diagnosis would change their lives forever and alter their parenting experience. **Theme 2**: Parents reported they were not given emotional support regarding the diagnosis. Parents experienced significant distress when medical decisions were imposed at this time.	Qualitative
19	Janvier et al. (2016) Canada	To examine parental goals/decisions, the length of life of their child and factors associated with survival.	Mixed methods	Descriptive statistics and thematic analysis	**Theme 1:** Parents identified goals that they felt would support them in their grief e.g., meet their child alive. **Theme 2:** Parents reported meeting some professionals who judged their decision to continue with the pregnancy.	Quantitative and qualitative as mixed methods study
20	Janvier et al. (2020) Canada	To investigate parental experiences with clinicians and to provide practical recommendations to clinicians to avoid conflict.	Mixed methods	Descriptive statistics and thematic content analysis	**Theme 1:** Trust identified by parents as crucial for supportive interactions. **Theme 2:** Parents reported certain disagreements with professionals related to the value placed on the child’s life.	Quantitative and qualitative as mixed methods study
21	Khalid et al. (2022) Malaysia	To explore the acceptance and usefulness of ACP discussions among parents of Malaysian children with bilateral cerebralsy palsy, potential sociodemographic, clinical, and ACP component variables related to parental acceptance.	Quantitative	Statistical analysis and Likert scale.	**Theme 1:** Parents who felt comfortable discussing EoL were more likely to discuss ACP. **Theme 2:** A proportion of parents were not aware that their child’s condition was life-limiting prior to survey. A minority of parents were not comfortable discussing EoL plans, however the reason for this was unclear.	Quantitative
22	Kiernan et al. (2022) Ireland	To explore the experience of healthcare professionals caring for children with NMLLCs across various healthcare settings in Ireland.	Qualitative descriptive study	Thematic analysis	**Theme 1:** Professionals provided emotional support for parents throughout trajectory and following child’s death described by being a supportive presence, listening ear, normalizing parental experiences, advocacy role, and developing relationships. **Theme 2:** Professionals expressed concern for the significant burden carried by parents with limited psychosocial support. They had concerns about their vulnerability. Communication across different settings reported as challenging.	Qualitative
23	Lord et al. (2020) Canada	To examine the experiences of ACP for children with medical complexity from the perspective of bereaved parents.	Qualitative	Thematic analysis	**Theme 1:** Relationships with palliative teams were considered important at the EoL. Parents advocated these services be introduced early in the trajectory. **Theme 2:** Parents reported that the ACP process was experienced as hurried. Parent advocated for hospital-based bereavement support.	Qualitative
24	Lövgren et al. (2016) Sweden	To explore experiences and wishes of bereaved parents concerning end-of-life care for their child with severe spinal muscular atrophy.	Quantitative	Descriptive statistics and content analysis	**Theme 1:** Findings suggested that parents’ wishes surrounding the circumstances of their child’s death were mostly achieved regarding saying goodbye to their child (92%), location of death (67%), and spending time with their child after death (90%). After the death, professionals supported parents to collect mementoes. **Theme 2:** A proportion of parents had negative experiences at the time of death that caused them further distress.	Qualitative
25	Sullivan et al. (2014) Australia	To examine parents’ views and experiences of end-of-life decision-making.	Qualitative	Thematic analysis	**Theme 1:** Doctors had discussed life forgoing treatments with all parents. Doctors who were supportive of parents as decision-makers were considered helpful. **Theme 2:** Some parents felt that judged when they made a decision to forgo treatment. These parents reported they were not offered palliative care or bereavement support.	Qualitative
26	Sullivan et al. (2015) Australia	To further elucidate findings on the nature and experience of parental involvement in decisions, and to clarify the ethical question of how decision-making should be approached with parents at end of life.	Qualitative	Thematic analysis	**Theme 1:** Parents regarded being facilitated to make EoL decisions as part of the parenting role. **Theme 2:** Parents who were not facilitated to make EoL decisions felt they had not fulfilled their parenting responsibilities.	Qualitative
27	Sullivan et al. (2019) Australia	This study explored the views and experiences of bereaved parents of acting on their decisions in end-of-life for their child.	Qualitative	Thematic analysis	**Theme 1:** Following the decision to forgo treatment, parents focused on the child living a non-medicalised, and preparing themselves for their child’s death. **Theme 2:** Parents reported having to reassert their EoL decision when professionals did not comply with their EoL plan.	Qualitative
28	Sullivan et al. (2020) Australia	To explore the aftermath of bereaved parents’ decision-making at the EoL.	Qualitative	Thematic analysis	**Theme 1:** Parents reported that making the EoL decision was helpful in their bereavement. Parents sought bereavement support from professionals they had a relationship with before the child’s death. **Theme 2:** Some parents had continuing concerns when professionals did not respect their decisions at the EoL, or when they interpreted the lack of follow-up support as professionals’ judging their decision-making.	Qualitative
29	Verberne et al. (2019) The Netherlands	To examine parental experiences and coping strategies when caring for a child receiving pediatric palliative care.	Qualitative	Inductive thematic analysis	**Theme 1:** Parents describe losses impacted on normal family life, privacy in the home, and of future dreams. Supportive interactions included having their feelings acknowledged and being able to tell their story. **Theme 2:** Uncertainty about disease progression caused feelings of uncertainty and lack of control, and parents reported they were not offered support to manage these feelings.	Qualitative
30	Walker et al. (2008) U.S.	To explore families’ overall experiences in the health care system after receiving a diagnosis of trisomy 18 with reference to the quality of their interaction with health care providers and to identify aspects of care associated with satisfaction.	Mixed methods	Content analysis	**Theme 1:** Parents identified that discussing the circumstances of the child’s death with medical professionals was helpful during the grieving process. **Theme 2:** A proportion of parents who continued pregnancy felt professionals judged them and did not offer medical interventions that may have increased the chance of survival. Parents experienced a lack of control over decision-making.	Qualitative as results focus on the interviews only
31	Wool et al. (2017) U.S.	To identify which quality indicators predict patient satisfaction with care in the prenatal setting when a foetus has been diagnosed with a life-limiting condition.	Quantitative	Statistical analyses	**Theme 1:** Satisfaction with care was associated with being facilitated to able to parent their baby and establish a bond, as well as bereavement support and collecting mementos. **Theme 2:** No information on the professionals’ practices as study reported on parents’ satisfaction with care.	Quantitative
32	Wool et al. (2018) U.S.	To examine quality indicators and how they influence satisfaction with care following the birth of an infant with a shortened lifespan.	Quantitative	Statistical analyses	**Theme 1:** The majority of parents had palliative support. Parents recognized the importance professionals listening to them, involving them in decisions, supporting choices to continue with pregnancy, and following through with care plans. Parents perceived that addressing bereavement was associated with satisfaction with care.	Quantitative
33	Zaal-Schuller et al. (2016) The Netherlands	To investigate the experiences of the parents and the involved physician during the end-of-life decision-making (EoLDM) process for children with PIMD.	Qualitative	Thematic synthesis	**Theme 1:** Doctors reported that shared decisions could support the grieving process. **Theme 2:** Parents queried if invasive treatments could have been avoided by engaging in EoL discussions when the child was stable. Some parents reported disturbing disagreements when they perceived the health system did not value their child the same as their treating doctor.	Qualitative
34	Zaal-Schuller et al. (2018a)	To address what elements do parents and physicians consider important for QoL in children with PIMD; How do parents and physicians incorporate QoL during EoLDM; and how they discuss QoL during EoLDM?	Qualitative	Thematic synthesis	**Theme 1:** Doctors understood the importance of involving parents in EoL decisions and agreed that QoL was an important factor in these decisions. **Theme 2:** Some parents feared that doctors would not intervene to save their child, they needed to show that quality of life was different when the child was well and not in hospital.	Qualitative
35	Zaal-Schuller et al. (2018b)	To investigate the involvement in the hospital of nurses in discussions with parents and physicians about end-of-life decisions for children with PIMD.	Qualitative	Thematic synthesis	**Theme 1:** Nurses felt parents only discussed their concerns about the child’s deterioration with them. Some parents consulted with these nurses before EoL discussions with doctor. All nurses had conversations with parents after the EoL discussion with doctor. **Theme 2:** Some nurses were not consulted during EoL discussions, despite having a supportive relationship with parents, and having responsibility for following through with the care plan.	Qualitative

ACP = advance care planning, SMA = spinal muscular atrophy, EoL = end of life, SNI = severe neurological impairment, TLD = treatment limitation discussion, NICU = Neonatal Intensive Care Unit, PPC = Pediatric Palliative Care, HMV = Home Mechanical Ventilation, PND = Prenatal Diagnosis, LTLLC = life-threatening or life-limiting condition, NMLLC = nonmalignant life-limiting condition, EoLDM = end-of-life decision-making, PIMD = Profound Intellectual and Multiple Disabilities, QoL = quality of life

* Paper excluded based on MMAT.

In the parent participant studies, 13 used qualitative methods. Of these, 2 involved parents, 6 bereaved parents, and the remaining 5 both parents and bereaved parents. Ten papers used a quantitative research design. Four of these included parents who had received a prenatal diagnosis and had opted for an induced termination or where the fetus had not survived. Of the remaining 6 papers, participants in 1 were parents, with 5 papers comprised of both parents and bereaved parents. Both mixed methods papers comprised parents and bereaved parent participants.

In papers reporting professionals’ views, 2 were qualitative and 2 quantitative. The remaining 3 papers were chart reviews. The final paper used mixed methods in conjunction with a retrospective chart review.

All studies analyzed were conducted during a 10-year period from 2012 to 2022 across several countries, including America, Canada, Europe, Australia, and Malaysia. Children’s ages ranged from 1 day to 17 years, with evidence of age clusters associated with the condition. Noting that studies focused on death and dying, the majority of discussions occurred later in the trajectory.

As noted above, it was an unexpected finding that these studies did not include explicit discussions of loss and grief, however analysis of findings relating to discussions of death and dying generated 2 themes relevant to understanding loss and grief in the context of LLSNI.

### Theme 1: Loss and grief is part of this context

This theme was developed in light of the unexpected finding that none of the papers identified explicitly reported on discussions between professionals and parents about aspects of loss and grief. Despite this, the analysis of these papers did isolate evidence that loss and grief is part of parents’ experience and professionals’ practice. All papers explicitly reported on discussions of death and dying, and most discussions related to medical interventions during the end-of-life phase. There was evidence of practice being attuned to parents’ anticipatory grief and bereavement needs, with findings in most papers suggesting a broad understanding of the grieving process.

Trust was key to parents’ perceptions and experiences that care received was compassionate and empathic. Several papers reported on types of supportive interactions and care that engendered trust. These included expressions of compassion and empathy, when professionals conveyed they wanted the best for the child, and respected parents’ expertise in their desire to protect and care for their child (Beecham et al. 2017; Lord et al. 2020; Zaal-Schuller et al. 2016). It was evident that professionals trusted parents knew their child best and would make the right decisions when relationships had developed over a longer illness trajectory. Furthermore, professionals who invested in relationships felt better equipped to understand parents’ perspectives and align with goals of care (Donovan et al. 2022; Hurley et al. 2021; Zaal-Schuller et al. 2016). In addition, knowledge of parental dynamics was perceived as helpful for determining parent readiness cues prior to the initiation of advance care planning discussions (Carr et al. 2022a). In this vein, a trusting relationship facilitated ongoing discussions with parents regarding quality-of-life issues as the condition progressed.

However, trust could also be established during a single conversation. Parents described experiences of interactions that supported trust when informed of their child’s diagnosis (Currie et al. 2016; Janvier et al. 2016; Kiernan et al. 2022), specifically the use of empathetic language, when information was imparted in a balanced manner and communicated messages of hope. Parents perceived these interactions as conveying their child’s life was viewed as meaningful and valuable despite their diagnosis. Parents reported feelings of shock, loss, and grief at this time, and understanding the implications of the diagnosis was a complex mental process (Carr et al. 2022a). They reflected that life was unalterably changed, their hopes and dreams for their child had disappeared and they experienced uncertainty about the future (Hurley et al. 2021). While the review uncovered no evidence of emotional support to help process these feelings, it was evident that nursing staff were attuned to the loss of the expected parenting experience at these times. In 2 studies, nurses were perceived to support parent-and-child bonding in challenging circumstances in the neonatal intensive care unit (Currie et al. 2016; Guon et al. 2014). In addition, accounts of professionals’ assisting parents to acquire new parenting skills to meet complex care needs were noted (Courtney et al. 2018; Janvier et al. 2020).

Several papers identified that prolonging the child’s life was of major concern to parents, but this had to be balanced with the overarching priority to maintain their child’s comfort (Bogetz et al. 2020, 2022, 2021; Zaal-Schuller et al. 2018a). Parents held on to this liminal space by accepting their situation, normalizing the experience, and suppressing grief-related emotions (Bogetz et al. 2020; Carr et al. 2022a; Verberne 2019). Professional support for their goals of care alleviated some of the pressure experienced by these competing demands. Since disease progression provoked anxiety and fear, the creation and accomplishment of goals appeared to help parents feel they had some control over certain aspects of the outcome (Verberne et al. 2019). In this vein, communication practices that were open, honest, and understandable helped their engagement in discussions and decisions about future care needs. While accepting the inevitable deterioration in their child’s condition required continual adaption, parents felt supported when professionals acknowledged the sacrifices they made.

Findings, however, suggested that parents experienced advance care planning discussions as simultaneously supportive and anxiety provoking. In one paper, the introduction of palliative care was perceived as a clear sign of disease progression and the next step in the child’s journey (Verberne et al. 2019). While papers identified differences in professional practices regarding the timing of these discussions, it was evident that the involvement of palliative care teams facilitated discussions about the child’s impending death, particularly when parents perceived the regular medical team to be uncomfortable discussing death (Carr et al. 2022a). It encouraged the contemplation of goals and wishes, memory-making, and greater attention to symptom management and quality of life issues (Donovan 2022; Lord et al. 2020). Legacy-making activities enabled parents to mentally prepare for their child’s end-of-life, acknowledged their parenting role, and enhanced their sense of control by affirming parents’ decision-making at the end-of-life (Beecham et al. 2017). While not reflecting a discussion with parents about loss, in one study professionals stated the involvement of parents in end-of-life decision-making facilitated the grieving process, showing an understanding of this issue while not explicitly addressing it with the parent (Zaal-Schuller et al. 2016).

Post-loss, caring for the child’s body was identified as something parents valued along with being able to spend time with their child to say goodbye (Lövgren et al. 2016). Integrating bereavement supports with end-of-life care by sign-posting sources of support was linked to increased satisfaction with care (Walker et al. 2008), although some parents advocated that bereavement care should ideally be delivered by the treating team or counsellors familiar with complex needs (Lord et al. 2020).

### Theme 2: Lack of recognition of loss and grief

While parents recognized that palliative care was challenging work, they felt the onus was on professionals to understand what they were going through, to express empathy and compassion, and adjust their practice accordingly. This contributed to the development of this theme.

Poor communication practices at the diagnostic stage were perceived to have implications for parental acceptance of the diagnosis and prognosis and set a tone for future encounters with professionals. Parents reported that the shock of the diagnosis impacted on their ability to process and understand what they were being told (Carr et al. 2022a). Parents reported feeling like “their world had fallen apart” (p. 29, Hurley et al. 2021) but received no support from healthcare professionals to process their emotions. Parents described being informed of their child’s diagnosis by professionals who used insensitive or derogatory language, as well as unfamiliar medical terms (Janvier et al. 2016, 2020). Years later, parents were able to recall the exact words used during such encounters. Other factors that contributed to perceptions that professionals lacked awareness of parents’ emotional distress included diagnostic information imparted in inappropriate settings, alone, and without specific information on the condition. Moreover, remarks overhead regarding potential survival or future abilities were perceived as a judgment on the value of their child’s life (Janvier et al. 2016). These troubling experiences were etched in parents’ memories and became part of their story.

Prognostic uncertainty gave rise to tensions within parent-professionals’ relationships when it was perceived that care delivered was not individualized to family needs (Bogetz et al. 2022; Heckford and Beringer 2014). Papers indicated that while parents preferred information to be delivered in a straightforward way, differences in personality and coping styles influenced preferences regarding the detail, timing, and nature of information imparted (Beecham et al. 2017). For example, some parents perceived there had been missed opportunities to plan for the end-of-life and were given false hope when positives were emphasized in treatment discussions, whereas others coped by prioritizing the “here and now” to normalize life and tended to avoid prognostic discussions (Bogetz et al. 2021). In addition, parents highlighted improvements in communication across various agencies involved in their child’s care would enhance the care received (Kiernan et al. 2022).

In total, 2 papers reported that bereaved parents viewed their involvement in end-of-life decisions improved bereavement outcomes (Carr et al. 2022a; Zaal-Schuller et al. 2018a). However, preparing for the end-of-life was also a time when unsettling disagreements could occur. In particular, discussions about withdrawal of treatment were perceived to be acrimonious when goals of care and values differed (Bogetz et al. 2021). In 2 studies, parents perceived that professionals’ candid descriptions of the child’s likely physical deterioration, as well as their repeated reminders of the child’s poor prognosis were used to influence their treatment decision-making (Beecham et al. 2017; Hurley et al. 2021). Notably, these studies also reported occasions when overwhelming emotions hampered parents’ ability to make important decisions that could affect treatment outcomes. Parents wanted professionals to take control at such times.

It was evident that the uncertain trajectory had an influence on the timing of advance care planning discussions. These discussions typically occurred in either hospital or hospice settings. Bereaved parents felt discussions should ideally have occurred earlier and regularly, whereas many proceeded a hospital admission following a deterioration in the child’s condition. Evidence of inconsistent access to advance care planning before the child’s death were identified in 3 studies (Carr et al. 2022a; Guerin et al. 2020; Heckford and Beringer 2014). Findings from one study indicated that where curative management was ongoing, professionals were unlikely to discuss the likelihood the child would die until close to death (Heckford and Beringer 2014). In this vein, professionals’ discomfort caused frustration to parents who sought to know what to expect when their child reached the end-of-life. While palliative care was perceived to give permission for discussions about end-of-life plans (Sullivan et al. 2019), in one study, palliative professionals expressed a view that the majority of medical teams failed to recognize the palliative care needs of children with SNI (Guerin et al. 2020).

Several papers identified the loss of long-standing medical relationships after death as a further significant loss (e.g., Lövgren et al. 2016). Parents expressed a view that since these professionals were now part of their child’s legacy, maintaining contact, post-loss, was important for their grieving process.

## Discussion

It was evident that parents were confronted with loss from the moment the devastating news of their child’s diagnosis was first imparted. The reality of being separated from their child at some point in the future provoked anxiety and fear, with parents engaging in a complex mental process to comprehend their situation and to cope with challenging emotions over time. Uncertainty in prognosis and trajectory were perceived to have both redemptive and unsettling consequences. On the one hand, it enabled parents to advocate to ensure their child’s needs were prioritized, to be a “good parent” in the time they had, and to live life to the full (Donovan et al. 2022). In this respect, parents perceived it as important that their child was treated as normally as possible. At the same time, reminders of their child’s shortened lifespan, whether subtle or obvious, prompted parents to reevaluate goals, and to engage in a deeply emotional process to adjust to their loss (Carr et al. 2022a). While parents demonstrated personal resilience in normalizing the profound challenges that confronted them (Verberne et al. 2019), there was evidence that professionals who showed empathy and compassion (Hurley et al. 2021) took time to understand the whole family’s needs (Beecham et al. 2017), and acknowledging their parenting role aided them to move forward (Lövgren et al. 2016). Findings suggested that parents turned to known and trusted professionals, who had intimate knowledge of what the family had been through to make sense of loss (Kiernan et al. 2022). Finally, involving parents in end-of-life planning, as well as encouraging their participation in legacy-making activities showed that professionals were attuned to what might be helpful to grieving parents after the child died (Donovan et al. 2022). However, in only a small number of papers were these practices explicitly linked to improved bereavement outcomes.

The importance of interactions with healthcare professionals, as well as symptom-management at the end-of-life has been shown to influence long-term parental grief (van der Geest et al. 2014). In the current review, parents of children with SNI reported mixed experiences of professional interactions, with particularly troubling experiences described during the diagnostic period. Parental distress resulting from these interactions appeared to linger and added to their loss (Lövgren et al. 2016). While reflecting on these events after the death, it is conceivable that parents may have experienced regret and unfinished business. While the literature on regret within pediatric populations is limited, regret in adult populations has been shown to be linked with reduced quality of life, problems with grief symptomatology, and depression (Holland et al. 2020). In this vein, the review highlighted certain practices that were seemingly undertaken to facilitate positive reflections regarding the care received after the death. Professionals who provided emotional and instrumental support in the home were uniquely placed to observe parents’ workload and the tremendous strain on parents to extenuate periods when the child was well. Parents’ attempts to make sense of disease progression by clarifying medical information or reviewing the child’s quality of life with known and trusted professionals appeared to have a buffering effect, and aid parental coping over time (Kiernan et al. 2022; Zaal-Schuller et al. 2018b). In bereavement, sense-making has been found to account for 15 times the intensity of parent’s distress than other variables such as gender, type of death, or length of time bereaved (Keesee et al. 2008).

While previous research has shown that preparation for end-of-life is helpful for dealing with anticipatory grief and facilitates the grieving process (D’Agostino et al. 2008; Kreicbergs et al. 2007), the review highlighted the tendency for discussions to proceed following a deterioration in the child’s condition (Carr et al. 2022b). Nevertheless, there was consistent evidence that the introduction of palliative care was associated with a greater focus on family-centered care (Donovan et al. 2022). Findings however, suggested that not all children had timely access to palliative services, if they received them at all (Guerin et al. 2020). A study by Price et al. found that different trajectories lead to inequitable provision, with services for nonmalignant conditions less well-developed compared to those for pediatric cancer patients (Price et al. 2012). These findings suggest the uncertain trajectory for children with SNI may contribute to missed opportunities for referrals to palliative services. The review highlighted that offering choice about location of care and death facilitated parents to maintain their parenting role to the end, through their involvement in decisions and the physical care of their child. Findings also evidenced professional engagement in legacy-making tasks. In bereavement, legacy activities play an important role in maintaining a relationship with the deceased post-loss (Klass et al. 2014), however such activities were not explicitly linked to bereavement outcomes in the papers reviewed. Therefore, it was unclear whether professionals, in actuality, understood the theoretical basis underpinning these practices. Finally, being facilitated to spend time with the dying child in private to say goodbye and attend to the body after death was valued by parents. Previously, Wijngaards-de Meij et al. (2008b) found that the opportunity to say goodbye was associated with lower levels of grief in parents. However, it seemed only those parents who accepted their child would eventually die were open to these types of interventions.

With regard to the quality of the research, the MMAT findings would suggest that the studies meet the minimum criteria. However across the research there are some consistent limitations including a reliance on convenience or purposive sampling, a concern about the generalizability of the samples based on medical and other criteria, a lack of access to consistent information where audits are conducted and a possible over reliance on mothers’ reports. While these are not fatal methodological flaws, they do limit the representativeness of the research. In terms of the quality of the review, the work done to adhere to international guidelines for scoping studies and the involvement of multiple researchers in screening and interpretation give confidence. However it is notable that the review was not able to answer the main research question as originally intended.

Overall, these findings point to major gaps in the evidence base and our understanding of professionals’ practice in this area, although it is possible that discussions on aspects of death and dying may have been richer and more nuanced than was reported. Nevertheless, the significant impact that professionals’ have in the lives of these parents is clear from the papers reviewed, together with first-hand knowledge of the losses faced during their child’s trajectory. Going forward, it is recommended that research focus on further exploration of professional views to elicit whether more can be done to enhance bereavement outcomes given the trajectory of children with life-limiting SNI. To this end, it would be important to elucidate the evidence for practice that aligns with or is at variance with current bereavement research. In addition, the views of parents regarding their support needs were not adequately captured in the findings reported and this needs further investigation given the implications for evidence-based practice. Finally, since this area causes enormous anxiety for professionals, identifying training and support needs would benefit practitioners.

## Conclusion

The review revealed major gaps in our understanding of professionals’ discussions of loss and grief within this population. Notably, most papers did not consider bereavement outcomes when reporting findings. Despite this, all papers discussed aspects of death and dying, and findings suggest that professionals implicitly provided support for grief in this context. The focus on the end-of-life phase provided limited scope for evidence of discussions earlier in the trajectory. Findings point to the possibility that prognostic uncertainty may contribute to less well-developed palliative services for children with SNI. Initial ideas to advance evidence-based knowledge have been highlighted.

## Supporting information

Brennan et al. supplementary material Brennan et al. supplementary material

## Supplementary material

The supplementary material for this article can be found at https://doi.org/10.1017/S1478951524001743.
