# Supplementary material for: Healthcare professionals’ discussion of loss and grief with parents of children with life-limiting severe neurological impairment: Findings from a scoping review
Source: Palliat Support Care. 2025 Feb 21;23:e58. doi: 10.1017/S1478951524001743 (PMC13166582; doi:10.1017/S1478951524001743)
Supplement: Brennan et al. supplementary material [file S1478951524001743sup001.docx]

**Healthcare professionals’ discussion of loss and grief with parents of children with life-limiting severe neurological impairment: Findings from a scoping review**

**Supplementary Material**

PsycINFO Database Search String:

“life limiting neurodevelopmental disabil*” OR “neurodevelopmental disorders” OR "life limiting condition" OR "life limiting illness" OR “complex care need" OR "progressive disabil*" OR "life-threatening condition” OR "chronic illness" OR “chronic disease” OR “disease progress*” OR “end of life” OR (DE “Disease Progression”) OR (DE “Critical Illness”) OR (DE “Chronically Ill Children”)

AND

child* OR kid* OR infant* OR toddler* OR pre-school* OR youth* OR “young person” OR adolescen* OR p#ediatric OR teen* OR student* OR pupil* OR ( DE "Preschool Students") OR (DE "Pediatrics”)

AND

parent* OR mother* OR maternal OR father* OR paternal OR famil* OR caregive* OR (DE "Parents") OR (DE "Mothers") OR (DE "Fathers") OR (DE "Family") OR (DE "Caregivers")

AND

Death OR Grief OR Loss* OR "palliati* care" OR dying OR coping OR bereave* OR griev* OR mourn* OR terminal* OR "child mortality" OR adaptat* OR “end of life care” OR (DE "Death and Dying") OR (DE "Grief") OR (DE "Coping Behavior") OR (DE "Palliative Care") OR (DE "Adaptive Behavior") OR (DE" Bereavement") OR (DE "Child Death")
